# The Origin of Biased Sequence Depth in Sequence-Independent Nucleic Acid Amplification and Optimization for Efficient Massive Parallel Sequencing

**DOI:** 10.1371/journal.pone.0076144

**Published:** 2013-09-26

**Authors:** Toon Rosseel, Steven Van Borm, Frank Vandenbussche, Bernd Hoffmann, Thierry van den Berg, Martin Beer, Dirk Höper

**Affiliations:** 1 Operational Directorate of Virology, Veterinary and Agrochemical Research Centre, Ukkel, Brussels, Belgium; 2 Institute of Diagnostic Virology, Friedrich-Loeffler-Institut, Greifswald-Insel Riems, Germany; Columbia University, United States of America

## Abstract

Sequence Independent Single Primer Amplification is one of the most widely used random amplification approaches in virology for sequencing template preparation. This technique relies on oligonucleotides consisting of a 3′ random part used to prime complementary DNA synthesis and a 5′ defined tag sequence for subsequent amplification. Recently, this amplification method was combined with next generation sequencing to obtain viral sequences. However, these studies showed a biased distribution of the resulting sequence reads over the analyzed genomes. The aim of this study was to elucidate the mechanisms that lead to biased sequence depth when using random amplification. Avian paramyxovirus type 8 was used as a model RNA virus to investigate these mechanisms. We showed, based on *in silico* analysis of the sequence depth in relation to GC-content, predicted RNA secondary structure and sequence complementarity to the 3′ part of the tag sequence, that the tag sequence has the main contribution to the observed bias in sequence depth. We confirmed this finding experimentally using both fragmented and non-fragmented viral RNAs as well as primers differing in random oligomer length (6 or 12 nucleotides) and in the sequence of the amplification tag. The observed oligonucleotide annealing bias can be reduced by extending the random oligomer sequence and by *in silico* combining sequence data from SISPA experiments using different 5′ defined tag sequences. These findings contribute to the optimization of random nucleic acid amplification protocols that are currently required for downstream applications such as viral metagenomics and microarray analysis.

## Introduction

The determination of complete viral genome sequences is a growing field in human, animal, and plant virology. Complete genome sequences and their exponential growth in public databases (roughly 1.5 million sequences representing more than 100 000 viral taxa in GenBank at the moment of writing of this manuscript) not only allow for a better understanding of virus evolution, molecular phylogeny (phylogenomics) and epidemiology, but also facilitate functional analysis of virus genes in comparison with other sequences in databases. Traditionally, viral genome sequencing approaches are based on amplification of overlapping genome regions followed by Sanger sequencing [1]. As a result, efficient sequencing approaches rely very much on prior sequence knowledge and are often focused on specific groups of viruses to allow for robust design of amplification primers (e.g.[2]). Viral isolates from highly divergent families or less frequently studied viruses often require a cumbersome approach for genome completion, partly because of the lack of sufficient available sequence information for robust primer design, and partly because of frequent need for primer walking and redesigning primers.

Next generation sequencing (NGS) technologies were developed to accommodate the need of higher sequencing capacity and lower cost per nucleotide for large genome sequencing projects (e.g. [3], reviewed in [4]). One main advantage of NGS platforms is the possibility to sequence DNA samples without any prior knowledge of the sequence for priming [3]. However, virus samples are typically loaded with host and contaminating nucleic acids. Enrichment for nucleic acids of interest is thus needed before these technologies become useful. This enrichment is often established by a targeted amplification of viral nucleic acids using virus or taxon specific primers. Examples include streamlined sequencing protocols for influenza A viruses [5], [6], classical swine fever virus [7] and foot-and-mouth disease virus [8]. These protocols allow completion of the viral genome(s) in a single experiment and provide sufficient sequencing depth to analyze the variability of RNA virus populations in a single sample (e.g. [9], [10]).

Truly sequence independent access methods to viral genomes have been developed in the field of viral discovery (reviewed in [11], [12], [13]). One of the most prominent technologies for random access to viral nucleic acids is Sequence Independent Single Primer Amplification (SISPA), and was originally described by Reyes and Kim [14]. Several modifications have been published, some including enrichment steps for viral nucleic acids using filtration and nuclease treatment (DNase SISPA, [15], [16]). After a filtration step and nuclease treatment, nucleic acids protected within virion particles are purified. The random primers used in subsequent complementary DNA production have a fixed amplification tag which is used in downstream PCR amplification. The resulting random amplicons are cloned and selected clones from this library are sequenced. Although the method was developed as a tool for identification of unknown viruses, Djikeng and colleagues [16] demonstrated its potential use for full genome sequencing of different model genomes, albeit at a high sequencing effort (100′s of colonies picked and sequenced for genome completion) and requiring a reasonable amount of virus (minimum 10^6^ virus particles). This method was also applied to the partial sequencing of a novel paramyxovirus in penguins [17], influenza viruses [18] and the identification of unknown viruses from experimentally infected mice [19].

Recent studies have combined random priming approaches with NGS to obtain sequence information from viruses. These include the identification of a novel mink astrovirus [20], the metagenomic analysis of Dengue virus infected mosquitoes [21], metagenomic analyses of viruses in human stool samples [22], and the control of live-attenuated vaccines [23] and other biological products [24].

Careful examination of the sequence data obtained in these studies shows a lack of homogeneous distribution of randomly generated sequence reads over the target genome [16], [21], [22], [23], [25] exposing one limitation of these random access methods for the determination of complete viral genomes. This does not only lead to gaps and areas of low coverage, but also to areas of exaggerated sequence depth that may result in bioinformatic artifacts during sequence assembly, even with high sequencing efforts. Although these shortcomings were noted before, no systematic experimental analysis of this phenomenon was undertaken.

The aim of this study was to elucidate the mechanisms that lead to biased sequence depth when using random amplification. Moreover, we sought to use gained knowledge to improve random amplification methods, aiming for high quality viral genomes at a limited cost without prior sequence knowledge. Avian paramyxovirus type 8 was used as a model RNA virus to investigate these mechanisms.

## Materials and Methods

### Viral sample

Avian paramyxovirus type 8 virus (APMV-8) was kindly provided by the German reference laboratory for Newcastle disease of the Friedrich-Loeffler-Institut, Greifswald - Insel Riems, Germany. APMV-8 has a linear single stranded RNA genome of negative orientation with a length of 15 342 bases. Virus propagation was performed in 8–10 day old specific pathogen free embryonated chicken eggs. Allantoic fluid was collected and the virus titer was determined by hemagglutination assays according to the Council Directive 92/66/EC (1992) using 1% chicken erythrocytes.

### Random access to viral nucleic acids using DNase SISPA

APMV-8 virions were purified starting from one milliliter (ml) of allantoic fluid. Centrifugation, filtration with 0.22 µM filters, nuclease treatment with 100 units DNase I, viral RNA extraction and sequence independent single primer amplification (SISPA) were performed as previously described [26]. Briefly, the RNA was denatured at 95°C for five minutes in the presence of random SISPA primer FR20RV-6N (5′-GCCGGAGCTCTGCAGATATCNNNNNN-3′, [15]) which was used in the double stranded complementary DNA (cDNA) synthesis reaction. This primer was composed of a random 6N oligomer tagged with a known sequence which was subsequently used as PCR primer binding-extension sequence with complementary primer FR20RV (5′-GCCGGAGCTCTGCAGATATC-3′). Purified, size selected (400–1 200 nucleotides [nt]) random PCR fragments were quantified with the Nanodrop-1000 spectrophotometer and used for the preparation of 454 sequencing libraries as described below.

### Optimization of DNase SISPA

The following modifications of the DNase SISPA protocol (on APMV-8) were performed to test whether annealing effects during cDNA synthesis and/or RNA secondary structures contributed to biased sequence depth.

#### RNA fragmentation

To test whether viral RNA secondary structures assisted in causing the unequal sequencing depth, we fragmented the extracted RNA according to the GS FLX Titanium cDNA Rapid Library Preparation Method Manual protocol (Roche, Mannheim, Germany, October 2009 Rev. Jan2010) starting from approximately 40 ng of viral RNA. RNA size distribution before and after fragmentation was measured using the RNA 6000 Pico chip (Agilent, Böblingen, Germany) on the Agilent 2100 Bioanalyzer. The fragmented RNA was used in SISPA under identical reaction conditions as described above.

#### Effect of the Primer-tag sequence

To check whether the primer tag sequence (designed for downstream PCR amplification) had an influence on the binding of the random primer along the genome during cDNA synthesis, alternative primer sequences were tested during first and second strand cDNA synthesis (summarized in Table 1). A primer with an alternative PCR amplification tag (K-6N, 5′-GACCATCTAGCGACCTCCACNNNNNN-3′, modified from [27]) was tested to investigate whether other regions in the genome would be preferentially targeted compared to the original FR20RV-6N (5′-GCCGGAGCTCTGCAGATATCNNNNNN-3′) primer. Primer K (5′-GACCATCTAGCGACCTCCAC-3′, modified from [27]) was used for downstream PCR amplification under identical reaction conditions as for FR20RV. Additionally, a 12N random sequence version was tested for both tag sequences in comparison to the 6N random sequence primers (FR20RV-12N, 5′-GCCGGAGCTCTGCAGATATCNNNNNNNNNNNN-3′ and K-12N, 5′-GACCATCTAGCGACCTCCACNNNNNNNNNNNN-3′).

**Table 1:** Summary of experimental conditions tested on APMV-8 and their assembly and coverage statistics.

		Assembly statistics			Coverage statistics						
Primer condition	RNA (F/N)*	n raw bases	n mapped bases	n raw reads (% mapped reads)	Min	Q1	Med	Q3	Max	IQR	% bases depth < 100 ×
**FR20RV-6N°**	N rep1	7 695 675	7 695 553	24 522 (100)	1	138	326.5	653	3 286	515	23.09%
	N rep2	7 695 161	7 695 029	31 372 (100)	1	139	333	734	2 109	595	19.56%
	F	7 695 068	7 694 932	26 683 (100)	1	149	307	650	2 458	501	17.31%
**FR20RV-12N**	N	7 693 226	7 692 897	26 770 (100)	1	253	394	647	1 609	394	2.11%
	F	7 693 689	7 693 254	27 565 (100)	1	268	435	672	1 418	404	3.25%
**K-6N°**	N rep1	7 695 878	7 695 698	25 834 (100)	1	140	271	579	3 096	439	15.72%
	N rep2	7 695 826	7 695 623	32 746 (100)	1	97	236	607	3 709	510	25.65%
**K-12N**	N	7 694 987	7 694 677	31 097 (100)	1	171	320	556	3 611	385	8.98%
**FR20RV-6N+K-6N**	N rep1	7 696 111	7 695 907	25 181 (100)	1	206	362	697	2 026	491	5.91%
	N rep2	7 695 231	7 695 032	32 115 (100)	1	178	371	724	2 208	546	7.68%
**FR20RV-12N+K-12N**	N	7 694 566	7 694 158	28 885 (100)	1	261	385	553	2 387	292	2.31%

° To rule out any random effects, the experimental conditions FR20RV-6N and K-6N were repeated independently starting from fresh virus culture aliquots of the same virus lot.

* RNA fragmented (F) or not fragmented (N).

### Sequencing

Purified, size selected (400–1 200 nt), random amplified DNA originating from the different random amplifications of APMV-8 was used to prepare sequencing libraries for the Genome Sequencer FLX (GS FLX; Roche, Mannheim, Germany). This was performed according to the manufacturer's instructions for Titanium Series reagents, using multiplex identifiers (MID) to identify the different libraries. The resulting libraries were sequenced with a GS FLX with Titanium Series reagents and run protocol (200 cycles).

### Data analysis

The sequence output file was sorted per sequencing library according their MID sequences. All raw sequence files were submitted to the Sequence Read Archive (SRA) under accession number SRP028373. Sequence reads were trimmed to remove the primer sequence including the random (6N or 12N) part as well as low quality ends. Non-APMV-8 specific reads were filtered out. Of each dataset approximately 7.7 Mb of raw data (≈ 500 × theoretical genome wide sequencing depth) were randomly picked to allow direct comparison between all conditions. Reference guided assemblies were performed relative to APMV-8/pintail/Wakuya/20/78 (GenBank: FJ215864) using the GS Reference Mapper software (version 2.6; Roche, Mannheim, Germany). Data output files were further processed with R ([28]; http://www.r-project.org/). To investigate if the 5′ specific amplification tag of SISPA primer influenced the sequence depth distribution, we mapped short sections of the amplification tag of the SISPA primers of increasing length adjacent to the random part of the primer along the genome sequence using R. Consensus sequences from each of the amplification strategies were compared in a clustalW alignment. Variant analysis was performed by mapping of all raw sequencing reads (complete datasets) of a certain condition relative to reference sequence APMV-8/pintail/Wakuya/20/78 (GenBank: FJ215864) using SeqMan NGen® version 3.0 (DNASTAR, Madison, WI, USA). After mapping, the reference was deleted and single-nucleotide polymorphisms were called. At polymorphic positions, we included a degenerate nucleotide in the consensus sequence if the minor nucleotide alternative was present in at least 30% of the sequence reads.

### Modeling secondary structure

RNAfold from the Vienna RNA Package version 2.0 ([29], http://www.tbi.univie.ac.at/RNA/) was used to predict partition function and base pairing probability matrix (dot plot). The pair probabilities were extracted and plotted in a mountain plot (Perl script mountain.pl, http://www.tbi.univie.ac.at/RNA/utils.html). This plot represents the secondary structure in a plot of height (number of base pairs enclosing the base at a certain position, i.e. a measure of local secondary structure complexity) versus position. As our SISPA protocol used 95°C to denature the RNA before cDNA synthesis and 50°C at first strand cDNA synthesis, we modeled the minimum free energy secondary structure of the RNA genome sequence at these temperatures.

### Positional genomic GC-content

Positional GC percentage was calculated with a sliding window of fixed size of 401 bp. The window was centered at a particular position and expanded 200 bp to either side of the center.

### Analysis of the virus specificity of the protocol

In order to establish the virus specificity of the protocol, all sequence data generated for each library were classified according to the species they belong to. To this end, a combination of BLAST and the GS FLX software suite (v2.6; Roche) was used to sort the reads. Subsequently, the percentages of reads identified as viral sequences were calculated as a measure of the virus specificity.

## Results

### Standard DNase SISPA results in highly variable sequence depth

**Figure 1:**
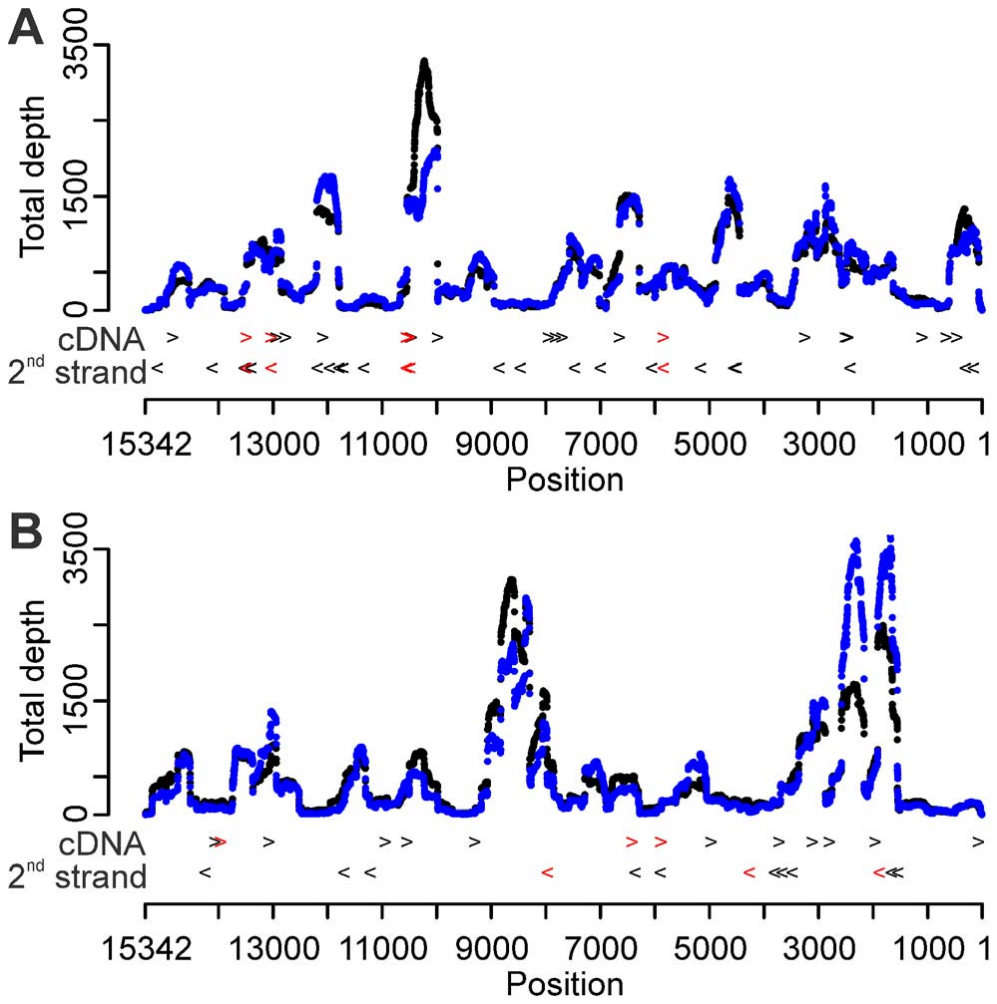
Distribution of sequence depth and enhanced annealing sites for two alternative 6N SISPA primers. (A) FR20RV-6N primer condition, repeat 1 (black) & 2 (blue). (B) K-6N primer condition, repeat 1 (black) & 2 (blue). The X-axis represents the position on the APMV-8 genome. Arrows to the right (>) represent potential enhanced annealing sites in the first strand cDNA synthesis direction, arrows to the left (<) in the second strand cDNA synthesis reaction. Black arrows symbolize enhanced annealing sites on the genome with five consecutive nucleotides in common with the 3′ end of the tag of the given SISPA primer; red arrows represent six or more common consecutive nucleotides.

Using the 6N SISPA primer FR20RV-6N [16], the complete coding sequence of APMV-8 could be determined using a reference assembly with approximately 7.7 Mb of raw data. This 7.7 Mb of raw reads were randomly picked from the complete dataset and corresponds to about 500 × sequence depth under the assumption of even sequence depth along the genome (Table 1). Despite the median sequencing depth of 326.5 ×, extreme variation in sequence depth (1 to 3 286 ×) was observed (Table 1; Figure 1 A, repeat 1). 23% of the genome nucleotides were covered less than 100 times, which we set as a minimum sequence depth to allow quantitative variant analysis (Table 1). An independent repetition starting from the same virus stock was made, producing a similar distribution pattern of sequencing depth (Figure 1 A, repeat 2). Apart from a lower maximum sequence depth (2 109 ×), the coverage statistics were reproducible (Table 1). The resulting consensus sequence was submitted to GenBank under accession number JX901129.

### RNA secondary structure and GC-content do not significantly influence sequence depth

**Figure 2:**
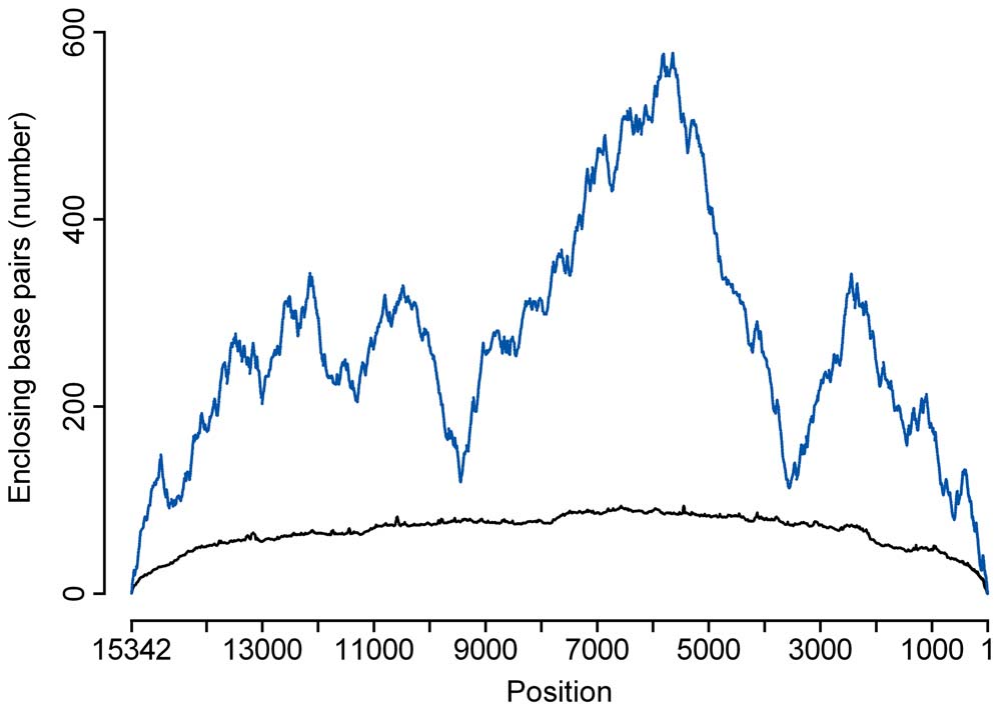
Mountain plot representation of viral RNA secondary structure. Based on base pair probabilities of genomic position. The Y-axis represents the number of base pairs enclosing the base at a certain position in the predicted RNA secondary structure (direct measure of secondary structure complexity). The X-axis represents the position on the APMV-8 genome. The equilibrium pair probabilities were predicted at 95°C (black) and 50°C (blue).

**Figure 3:**
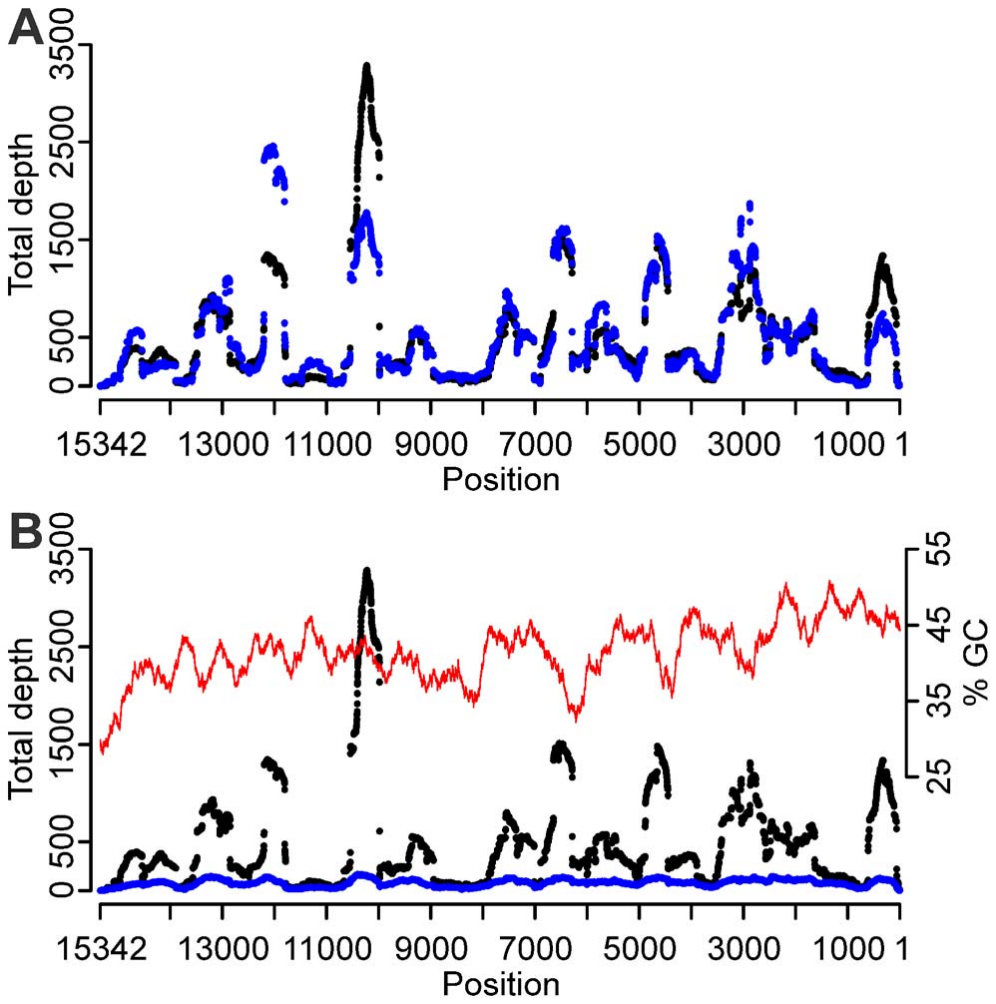
Effect of RNA fragmentation and GC-content on sequence depth distribution. (A) Fragmented (blue) and unfragmented (black) RNA, random amplified with the FR20RV-6N SISPA primer. The X-axis represents the position on the APMV-8 genome. (B) Total (black) and unique (blue) depth distribution of the FR20RV-6N SISPA primer condition. At unique depth, reads with identical starts are omitted. Also positions GC-content (window size 401) of the APMV-8 genome is displayed (red).

We could find no evidence of an overall correlation between areas below average sequence depth and global secondary structure of the viral RNA at denaturation and annealing temperatures (Figure 2 compared to Figure 1). However, the most complex region in the viral RNA secondary structure model (peaking approximately at position 6000) coincides with a region of low coverage under all conditions tested. To investigate the effect of RNA secondary structure experimentally, we compared libraries obtained from fragmented and non-fragmented RNA. The fragmentation of the RNA was successful with a shift of median RNA fragment size from 1 400 nt for non-fragmented RNA to 400 nt for fragmented RNA (RNA 6000 Pico chip results on the Agilent 2100 Bioanalyzer). To allow comparison with other conditions, we used approximately 7.7 Mb of raw data in the reference assembly. The coverage statistics were similar to the data obtained from non-fragmented RNA versus fragmented RNA (Table 1, FR20RV-6N and FR20RV-12N), and the distribution of coverage along the genome did not change (Figure 3 A).

The unique depth, roughly described as depth where reads with identical starts are omitted, did not show extreme variability over the genome (Figure 3 B).

We found no clear correlation between the GC-content (calculated with a shifting frame of size 401) and sequence depth (Figure 3 B; Spearman rank correlation coefficient of repetition 1∶0.09, P-value <0.005; repetition 2∶0.06, P-value <0.005).

### Fixed SISPA primer amplification tag sequence induces biased annealing

To analyze if the 5′ specific amplification tag of SISPA primer FR20RV-6N influenced the sequence depth, we mapped stretches of nucleotides adjacent to the random part of the primer along the genome sequence. The first one, two, three and four consecutive 3′ nucleotides of the amplification tag sequence occurred frequently along the genome (data not shown). Mapping of the possible annealing events on the APMV-8 genome during first strand or second cDNA strand synthesis aided by five, six, or even seven consecutive 3′ nucleotides revealed a clear correlation with the variability of the sequence depth (arrows on Figure 1 A). The six-nucleotide-enhanced annealing sites (red arrows) were found at the positions 5 843, 10 487, 10 549, 13 031, 13 490 along the APMV-8 genome. They were found both in first and second strand cDNA synthesis direction because of the palindromic nature of this sequence. Position 10 487 in the first strand direction and position 13 031 in the second strand direction even had 7 nucleotides in common with the 3′ end of the tag sequence. This resulted in an extreme increase of sequence depth in the adjacent regions (Figure 1 A). The combination of first strand annealing positions with some adjacent second strand positions located in antisense direction were associated with pronounced peaks in sequencing depth in both independent SISPA-NGS repetitions. In addition, a higher density of possible annealing sites resulted in more pronounced peaks in sequence depth (Figure 1 A; data not shown for shorter stretches). In contrast, genomic areas with a low density of enhanced annealing sites had lower sequence depth. It is worth noting that although region 12 851–13 483 had the highest density of potential annealing sites (stretches ranging from 3 to 7 nucleotides) of the whole genome, this region did not have the highest depth of the genome.

### Optimization of DNase SISPA annealing

In order to experimentally confirm the primer annealing bias and to improve the randomness of the SISPA protocol, we repeated the SISPA library preparation with modified conditions. These modifications included (a) a random annealing sequence extended from 6 to 12 nucleotides, (b) an alternative primer amplification tag sequence, and (c) the combination of the two primers with alternative tag sequences. The amount of raw data used in all assemblies was again kept at about 7.7 Mb to allow direct comparison between all conditions.

#### Extended random annealing part of the SISPA primer reduces bias

**Figure 4:**
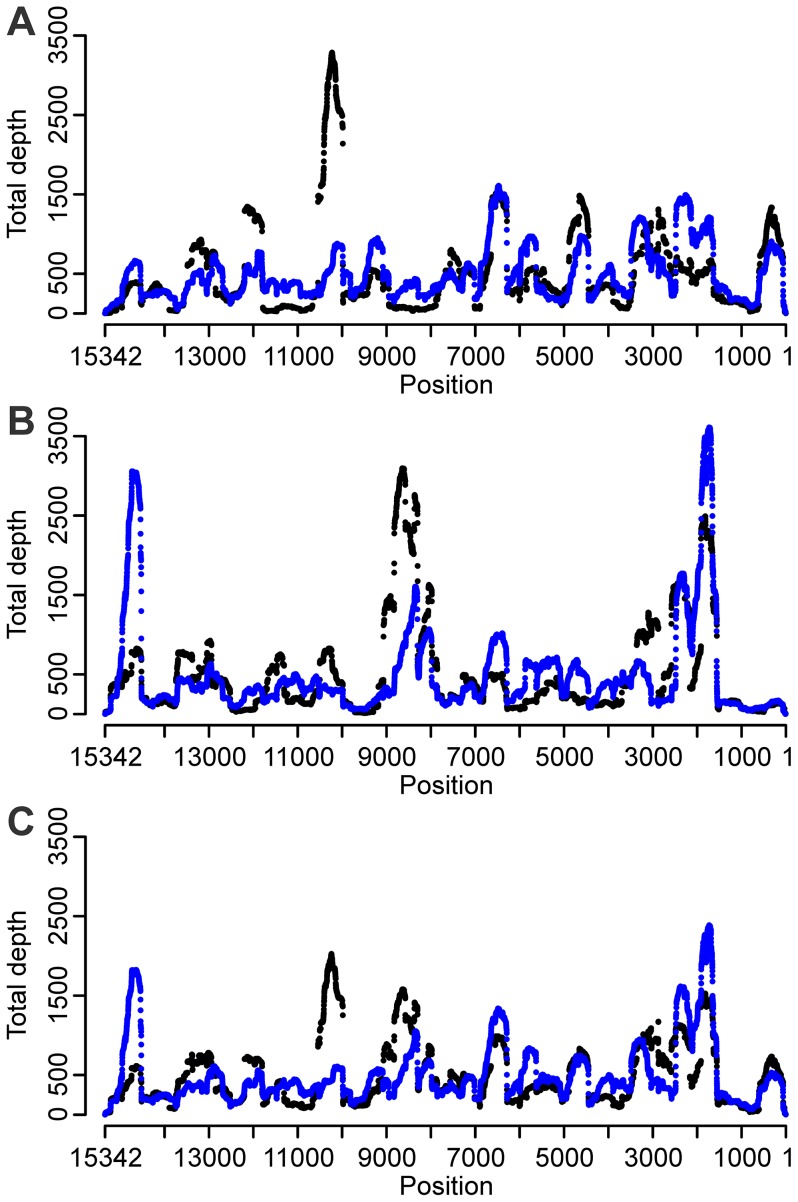
Effect of the SISPA primer sequence (random part and tag) on sequence depth distribution. (A) FR20RV-6N (black) and FR20RV-12N (blue). (B) K-6N (black) and K-12N (blue). (C) *In silico* combined conditions “FR20RV-6N+K-6N” (black) and “FR20RV-12N+K-12N” (blue).

In order to minimize the impact of the tag sequences on primer annealing we extended the random part of the primer while keeping the tag sequence constant. The 12N oligomer condition was performed on both non-fragmented and fragmented RNA. The use of a 12N SISPA primer resulted in a lower maximum depth, to the advantage of a higher first quartile and median sequence depth (Table 1). The number of genomic bases with depth less than 100 × was almost negligible (2.11%) compared to the 6N oligomer conditions (23.1%). The number of bases with depth above 500 × stayed the same, but extreme heights disappeared (Figure 4 A). A decreased interquartile range (IQR, Table 1) indicated more homogenous distribution of sequence depth across the genome. Increases and decreases in sequence depths could still be correlated with annealing influence of the amplification tag, but the effect was reduced compared to the 6N oligomer condition resulting in a more equally distributed sequence depth over the genome (Figure 4 A). Again, fragmentation of the RNA prior to cDNA production did not seem to exert any significant effect (Table 1) and no clear correlation between GC-content and sequence depth was found (Spearman rank correlation coefficient: 0.02, P-value <0.005).

#### An alternative amplification tag sequence relocates bias

Using a random 6N oligomer with an alternative amplification tag sequence, K-6N, the median and Q3 sequence depth were lower compared to sequences resulting from SISPA primer FR20RV-6N (Table 1). Again, highly variable sequence depth was obvious with peaks up to 3 709 × coverage and a high proportion (15.72%) of genomic positions with depth less than 100 × (Table 1). The use of an alternative amplification tag relocated the areas of extreme coverage to other genomic areas (Figure 1 B, repeat 1). When mapping the possible annealing bias introduced by the alternative amplification tag sequence to the APMV-8 consensus sequence, the correlation between strong annealing sites (of five, six, seven and now even eight 3′ nucleotides of the tag sequence) and coverage extremes was confirmed for this alternative tag sequence (arrows on Figure 1 B). The six nucleotide tag sequences (red arrows) were found at the positions 5 888, 6 416 and 13 957 along the APMV-8 genome in first strand cDNA synthesis direction and at positions 1 886, 4 271 and 7 966 in the second strand cDNA synthesis direction. Position 5 888 had seven nucleotides in common with the alternative tag sequence, position 7 966 even eight nucleotides. The high coverage peak in region after position 7 966 could be explained by annealing bias. On the other hand, no extreme coverage depth was visible near position 5 888. An independent SISPA repetition was performed from the same virus stock and confirmed our general findings (Table 1; Figure 1 B, repeat 2). 25.62% of genomic positions had a depth less than 100 ×. The maximum sequence depth of the peak observed in region 2 166–2 580 differed substantially between the two repetitions (repeat 1∶1 716 ×; repeat 2∶3 610 ×).

When comparing the number of enhanced annealing sites on the APMV-8 genome using primer K-6N and FR20RV-6N, we observed fewer enhanced annealing sites at K-6N (arrows on Figure 1 B compared to 1 A). This could contribute to the lower median depth of this K-6N condition (repeat 1∶271 ×; repeat 2∶236 ×) compared to the FR20RV-6N condition (repeat 1∶327 ×; repeat 2∶333 ×).

Using a 12N SISPA primer with identical amplification tag to K-6N (K-12N), the Q1 and median depth was increased, and both the IQR and number of positions with a depth of less than 100 × was decreased compared to the K-6N condition (Table 1). The extremes in the coverage plot were respectively flattened or raised, but the effect was less pronounced compared to the FR20RV-12N SISPA primer (Figure 4 B). The sequence depth extreme of 3 709 × in region 1 560–2 210 of the genome (Figure 4 B) might be explained by a remaining annealing bias effect as this region had the highest concentration of enhanced annealing places of 3 and 4 common nucleotides with the 3′ end of the primer tag, especially in the second strand direction (data not shown). A second unexpected observation was the peak at the 3′ end (14 500–15 000) which had increased enormously compared to the 6N oligomer condition. Moreover, this region did not show a concentration of enhanced annealing places. For logistical reasons, we were unable to perform an independent repetition of the K-12N condition for confirmation.

#### Combined primer sets reduce bias in silico

Because of the use of an alternative amplification tag sequence resulted in relocation of areas where enhanced annealing induced sequence depth peaks, we tested whether combined libraries from two different SISPA primers might result in a more homogenous sequence depth distribution along the entire genome. We modeled this possibility by performing a combined APMV-8 reference assembly with equal amounts of raw sequence data (each about 3.8 Mb; reads were randomly picked) resulting from the libraries produced using 6N SISPA primers FR20RV-6N and K-6N. As each of these libraries was repeated independently, we modeled two combined libraries resulting from the two repeats of the respective libraries (Table 1, Figure 4 C). Combining the two first repeats resulted in improved coverage statistics (increased Q1 and median depth, and decreased number of bases with depth < 100 ×) compared to the single SISPA primer conditions alone (Table 1). Combining the two second repeats confirmed this more equal distribution. The decreased IQR indicated a better distribution of depth which was also visible on the sequence depth plot. A similar combined assembly using the 12N SISPA primer conditions (FR20RV-12N and K-12N) resulted in an improvement of the coverage statistics of the stand-alone K-12N data (Table 1, Figure 4 C). Interestingly, this condition had the lowest IQR, with 50% of the genome positions having a coverage depth in between 261 × and 553 × (Table 1). However, combination of the two primer sets in a single reaction during reverse transcription/second strand cDNA synthesis/PCR repeatedly failed, probably due to interaction between the 2 different SISPA primers. Primer purification after 1^st^ and 2^nd^ cDNA strand synthesis would most likely solve this problem.

#### DNase SISPA-NGS has high sequence fidelity and allows reliable variant calling

When aligning the consensus sequences of the tested reaction conditions we did not observe any sequence differences. We looked for variation in the largest available dataset (FR20RV-12N condition) making use of all raw data. Only variant positions with depth >100 × and a percentage of minor nucleotide >10% were examined to allow reliable variant analysis (Table 2). In comparison we also evaluated variability in K-12N condition. Despite a variation in average depth between the two conditions (FR20RV-12N: 2 300 ×; K-12N: 1 158 ×), we observed a very similar variation ratio at the variant positions. For example at genome position 3 279 there was a variant calling of 20% ‘G’ and 80% ‘A’ in both FR20RV-12N (depth: 5 463 ×) and K-12N (depth: 1 378 ×). We found only big differences between conditions when the position was part of a homopolymer stretch (typical base-call errors caused by the 454 pyrosequencing technology). For example, position 2 102 had as major nt ‘A’ and as minor nt ‘G’. At the FR20RV-12N condition the minor percentage was 11% against 35% at K-12N. This position is preceded by 4 A's and followed by 3 G's. Overall, these data show that sequence variants present in at least 10% of the reads can be reproducibly identified using DNase SISPA-NGS.

**Table 2:** Variant calling in the 12-mer datasets at genome positions with depth larger than 100 × (except for position 31 in the K-12N condition) and minor nucleotide percentage of at least 10%.

position	condition	depth	major nt	minor nt	% minor
**31**	FR20RV-12N	366	C	T	28%
	K-12N	63	C	T	28%
**1 908**	FR20RV-12N	4 500	G	A	20%
	K-12N	5 279	G	A	22%
**2 102**	FR20RV-12N	3 946	A	G	11%
	K-12N	1 110	A	G	35%
**3 188**	FR20RV-12N	5 216	A	G	19%
	K-12N	1 073	A	G	19%
**3 279**	FR20RV-12N	5 463	A	G	20%
	K-12N	1 378	A	G	20%
**4 972**	FR20RV-12N	1 123	A	G	17%
	K-12N	515	A	G	13%
**6 526**	FR20RV-12N	6 535	G	A	19%
	K-12N	2 136	G	A	17%
**6 705**	FR20RV-12N	4 039	G	A	18%
	K-12N	1 357	G	A	15%
**7 760**	FR20RV-12N	1 481	G	A	20%
	K-12N	321	G	A	17%
**7 890**	FR20RV-12N	1 396	C	G	41%
	K-12N	724	C	G	34%
**7 895**	FR20RV-12N	1 457	G	A	11%
	K-12N	756	G	A	11%
**8 055**	FR20RV-12N	990	A	G	26%
	K-12N	214	A	G	14%
**12 803**	FR20RV-12N	2 652	A	C	33%
	K-12N	1 026	A	C	32%
**13 410**	FR20RV-12N	1 797	T	G	39%
	K-12N	872	T	G	38%
**14 913**	FR20RV-12N	1 611	A	G	18%
	K-12N	2 786	A	G	17%
**15 155**	FR20RV-12N	484	A	T	20%
	K-12N	430	A	T	28%

### DNase SISPA-NGS is highly specific for viral sequences

A metagenomics analysis of all sequenced raw data showed that all studied conditions were highly specific for APMV-8 RNA (Table 3). The proportion of APMV-8 specific sequences ranges from 87.7% using SISPA primer FR20RV-12N condition to 95.8% using SISPA primer K-6N (repeat 1). The remaining fractions contained almost no contaminating host or bacterial sequences, and most of the unassigned sequences in all datasets could be attributed to unknown sequences (most likely technical or sequence database artifacts). The protocols based on 12N SISPA primers were slightly less specific to the advantage of the eukaryotic/host sequences compared to the 6N primers.

**Table 3:** Metagenomic analysis of all sequence raw data from the different studied conditions.

Condition	Used reads	APMV-8	Bacteria	Eukaryotes	Unassigned
**FR20RV-6N, repeat 1**	90 137	94.99%	0.09%	0.31%	3.72%
**FR20RV-6N, repeat 2**	69 860	94.40%	0.58%	0.81%	3.62%
**FR20RV-6N, fragmented RNA**	80 473	93.61%	0.34%	1.13%	4.34%
**FR20RV-12N**	147 004	87.73%	1.00%	2.45%	6.35%
**FR20RV-12N, fragmented RNA**	133 895	88.87%	0.79%	2.35%	6.40%
**K-6N, repeat 1**	141 400	95.75%	0.15%	1.10%	2.69%
**K-6N, repeat 2**	116 494	93.84%	0.44%	0.86%	4.34%
**K-12N**	80 258	91.20%	0.42%	2.52%	3.87%

**Figure 5:**
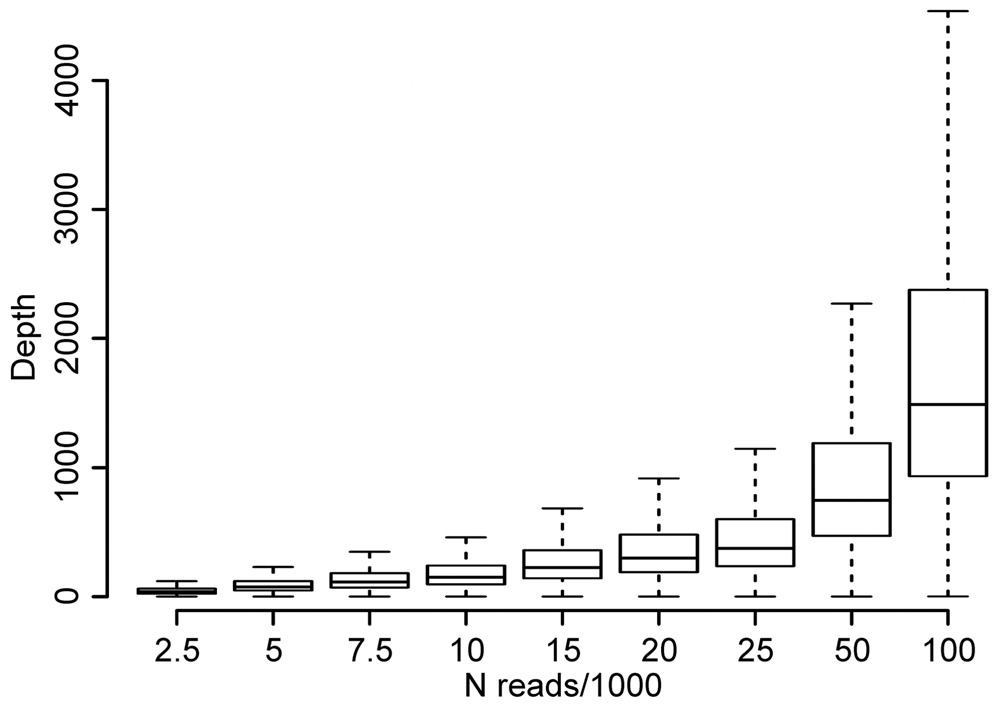
Effect of increasing sequencing effort on coverage statistics. Reads were randomly picked from the largest dataset (FR20RV-12N). This random sampling was repeated 10 times and averages of coverage statistics are displayed in boxplots.

The efficient targeting of the protocol enables sequencing complete viral genomes with very little sequencing effort. To investigate the relation between sequencing effort and sequence depth, we randomly picked increasing amounts of reads from the largest available dataset (FR20RV-12N) and looked at its coverage statistics. This random sampling was repeated 10 times and averages are displayed in Figure 5. As expected, the median depth increased linearly with an increasing sequencing effort. We observed also a higher IQR for higher sequencing efforts, which indicated a higher variation of sequence depth (Figure 5). In summary, it was possible to obtain nearly complete (99.9%) high quality genome sequences missing only a few nucleotides at the genome extremities using as few as 2 500 reads.

## Discussion

Sequence Independent Single Primer Amplification [14], also known as “random PCR”, is one of the most prominent random access techniques to viral nucleic acids. The method provides an efficient enrichment in viral nucleic acids, avoiding excessive host and other contaminant sequence reads [15], [16]. The metagenomics analysis of our data shows that the enrichment procedure for viral nucleic acids is highly efficient, while being applicable to any virus genome and requiring no prior sequence knowledge. A current limitation of the random access protocol described here is the need for sufficient virus material. Although combination of SISPA with NGS makes the protocol more sensitive, full genome sequencing of low titer samples directly from clinical material may be difficult as we recently discussed [30]. Virus genome specific amplification strategies may be better suited to increase the applicability to field samples for known viruses (e.g. [6]). Additionally, the method requires virion protected viral nucleic acids and as such is not applicable to cellular forms of viral nucleic acids (latent infections).

When combining random amplification with NGS, its tendency to create a biased distribution of sequence depth becomes evident (e.g. [22], [23]). Extreme variability in sequence depth does not only imply problematic regions of low coverage, also areas of exaggerated sequence depth compared to the median coverage may result in bioinformatic artifacts during data analysis such as *de novo* contig assembly and variant analysis. The tendency of the 454 technology to produce duplicate reads as a result of multiple beads present in the same micro-reactor during emulsion PCR [31], [32] alone cannot explain the dramatic differences between the coverage plots based on the total depth and those based on unique depth. Our study is the first to formally analyze the possible causes of this sequence depth variability.

Victoria and colleagues [22] suggested that regions with overrepresented sequencing depth are associated with annealing bias of the used primer, while secondary structure may result in areas of low sequencing depth. Formally modeling secondary structure and annealing factors on the one hand and using experimental data on the other hand, our study identified annealing bias as the main cause of the overrepresented regions after performing random priming in the SISPA method. Annealing of the random 3′ end of SISPA primers seems to be locally enhanced when 3, 4, 5, 6, 7 or even 8 nucleotides from their specific 5′ amplification tag (20 nucleotides in length) designed for PCR amplification assist the random oligomer part of the primer in annealing during the first strand and/or second strand cDNA synthesis. This results in regions of exaggerated sequence. The importance of this highly reproducible annealing effect seems to depend on the length of the complementary region between the primer and the genome and the proximity of first strand and second strand enhanced annealing positions. Going beyond a theoretical mapping of these annealing effects, our data show that the majority of sequence depth bias can be explained by this effect.

Using a different specific amplification tag sequence, we observed a clear shift of the coverage extremes to other genomic regions caused by the shift of possible enhanced annealing sites. Again, the coverage depth was very variable, but the median depth was lower compared to original used primer. This is probably due to the lower number of potential strong annealing places on the APMV-8 genome for the alternative random primer K-6N. This confirms that the distribution of possible enhanced annealing sites depends on the SISPA primer amplification tag sequence and on the target genome sequence, which implies possible differential annealing dynamics when targeting other virus genomes.

Using any of these SISPA primers, regions in the genome existed where predicted enhanced annealing sites did not result in extreme sequence depth, for example the region around positions 6 416 and 5 888 at the K-6N primer condition have respectively 6 and 7 nucleotides in common with the tag sequence. Similarly, the region around position 5 843 at the FR20RV-6N primer condition has high similarity with the tag sequence. However, these predicted enhanced annealing sites do not seem to coincide with increased sequence coverage in this region. When looking at the RNA secondary structure mountain plot at first strand cDNA annealing temperature (Figure 2, 50°C) we see that these positions are located in the most complex region of the genome. It should be noted that we started our first strand cDNA synthesis step with a denaturation at 95°C for a better removal of RNA secondary (Figure 2, 95°C) and tertiary structures. Nevertheless, it could be that the RNA locally refolds at lower temperature during cDNA synthesis. In addition, extreme coverage was also observed in regions which did not show a high concentration of enhanced annealing sites, for example genome region 14 500-15 000 at the K-12N primer condition. This region had a very low GC-content and no complex RNA structures, but did not yield high coverage when targeted with primer K-6N. In none of the conditions, a clear overall correlation was found between GC% and sequencing depth (Spearman rank correlation coefficient was always between −0.15 and 0.09, P-value <0.005). These observations show that, although being a major correlate of sequence depth, annealing bias is not the only factor contributing to variations in sequence depth. These sequence coverage variations that remain unexplained by SISPA primer annealing/extension bias highlight the complexity of annealing and extension dynamics which can be locally influence by multiple interacting factors such as RNA secondary structure, GC content and oligomer length. Victoria and colleagues [22] suggested already that complex RNA secondary structures contributed to regions with problematic sequence depth. We did not observe a change in distribution of sequence depth after fragmentation of the viral RNA for the studied avian paramyxovirus genome. Although the global secondary structure of the viral RNA is thus successfully fragmented, it cannot be excluded that local RNA folding may still have influenced primer annealing. However, we could not further fragment the RNA in order to assure compatibility with the RNA sequencing workflow.

These findings confirm earlier observations by Wong and colleagues [33]. That study used SISPA amplification in the context of a pathogen detection DNA microarray. In initial experiments using random priming amplification to identify pathogens they observed frequently incomplete hybridization of the pathogen genomes marked by interspersed genomic regions not detected by the probes that could not be explained by sequence polymorphisms, probes GC content and genome secondary structure. The composition of the SISPA primer tag had a significant impact on the efficiency of viral genome amplification – as suggested in our study. Using an algorithm to optimize primer sequences for uniform amplification efficiency across the viral genomes included in the DNA array, they managed to increase the sensitivity of pathogen detection of their microarray.

Previous studies have used different lengths of the 3′ random annealing part of the SISPA primer. Examples include hexamer [16], [17], [20], [26], [30], [34], [35], [36], [37], [38], octamer [19], [22], [23], [39], [40], [41], [42], nonamer [24], [43], [44], [45] and decamer random 3′ annealing parts [46]. Stangegaard and colleagues [47] studied the impact of different random primers (without 5′ specific amplification tag sequence) on the yield and quality of synthesized cDNA, concluding that reverse transcription using random pentadecamer primers increases yield and quality of resulting cDNA compared with hexa- and nonamers. In our study, we compared SISPA-primers with hexamer and dodecamer random annealing stretches. Theoretically, the number of possible annealing sequences for a 12N primer is 4^12^ ( = 16 777 216), compared to 4^6^ ( = 4 096) for a hexamer. In our study, increasing the random part of the SISPA primer from 6N to 12N reduced the amplification tag induced sequence depth bias to the advantage of the regions with problematic coverage.

We combined two factors affecting the distribution of sequence reads over the genome in a single assembly: (a) alternative SISPA primers are biased to alternative genome regions, and (b) longer oligomer-based SISPA primers tend to have a more equal distribution. The combination of data from libraries resulting from two different 12N SISPA primers (FR20RV-12N and K-12N) resulted in the best distribution of sequence reads over the APMV-8 genome. We suggest that in future efforts (using DNase SISPA for the determination of complete viral genomes), a combination of a longer random annealing part (e.g.12N) and the combined assembly of data resulting from libraries amplified with alternative amplification tags, may result in an improved homogeneity of sequence depth distribution over the genome. Ultimately, a more homogenous sequence depth distribution reduces the sequencing effort (and thus cost) needed for genome completion. In this study, 2 500 GS-FLX titanium reads were enough to sequence 99.9% of the APMV-8 viral genome with a median coverage of 38.3 ×. We have shown that median coverage increased with the increasing sequencing effort. 7 500 reads covered the full genome with a median depth of more than a 100 ×. This confirms our previous experience that only about 5 000 GS-FLX titanium reads from a library of SISPA amplified viral RNA was enough in determining the complete genome of uncharacterized avian paramyxoviruses [26]. It should be noted that amplified viral stock was used in this study.

Next generation sequencing is becoming increasingly accessible to laboratories, both through the evolution of sequencing platforms and chemistries and through the increasing availability of sequencing service providers. Combined with opportunities to multiplex samples during sequencing, this technology is now evolving towards a cost-effective methodology for genome sequencing. Generic, sequence independent access methods such as optimized DNase SISPA may facilitate access to viral genome sequences, without the need for prior sequence knowledge. In addition, our findings may be of value to other technologies requiring random nucleic acid amplification such as DNA microarrays.
